# Evaluation of thrombin generation in dogs administered clopidogrel

**DOI:** 10.3389/fvets.2023.1194242

**Published:** 2023-08-23

**Authors:** Kaitlyn Rank, Alex M. Lynch, Laura K. Ruterbories, Ronald H. L. Li, Yu Ueda

**Affiliations:** ^1^Department of Clinical Sciences, College of Veterinary Medicine, North Carolina State University, Raleigh, NC, United States; ^2^Department of Surgical and Radiological Sciences, School of Veterinary Medicine, University of California, Davis, Davis, CA, United States

**Keywords:** coagulation, platelet inhibitory, antithrombotic, therapeutic monitoring, canine

## Abstract

**Introduction:**

The antiplatelet effect of clopidogrel can vary between patients. A modified thromboelastography (TEG) protocol (TEG-Platelet Mapping assay^®^ [TEG-PM]) can be used for clopidogrel monitoring but is not widely available. Thrombin generation (TG) assays could offer a novel alternative. The main objective of this pilot study was to assess TG assay variables (lag time, peak, endogenous thrombin potential [ETP]) in dogs before and after 7 days of clopidogrel administration and compare with TEG-PM variables (maximum amplitude [MA]-ADP and percentage (%) inhibition).

**Methods:**

Six healthy mix-breed dogs were enrolled in this pilot study. Blood samples for platelet count, TG assays, and TEG-PM were obtained at two time points, corresponding to baseline, and after 7 days of clopidogrel administration (mean 2.3 +/− 0.3 mg/kg PO q24 hours). Data were then compared with a Student’s *t*-test.

**Results:**

There was no significant change in TG assay variables performed on platelet poor plasma after 7 days of clopidogrel administration: lag time (Day 1: 1.8 +/− 0.2 min, Day 7: 1.8 +/− 0.2 min, *p* = 0.42); peak (Day 1: 76 +/− 7 nM, Day 7: 72 +/− 10 nM, *p* = 0.49); and ETP (Day 1: 399 +/− 27 nM*min, Day 7: 392 +/− 32 nM*min; *p* = 0.49). There were significant changes in TEG MA-ADP (Day 1: 19 +/− 8 mm, Day 7: 9 +/− 6 mm, *p* = 0.04) and % inhibition (Day 1: 58 +/− 27, Day 7: 99 +/− 0.3, *p* = 0.02).

**Discussion:**

Clopidogrel administration did not lead to changes in TG assay variables performed on platelet poor plasma samples, despite concomitant changes in TEG-PM variables consistent with platelet inhibition. Based on this pilot study, thrombin generation performed on platelet poor plasma may not be a useful antiplatelet monitoring tool in dogs.

## Introduction

Thromboembolic disease is an important contributor to morbidity and mortality in dogs (1–5), with clopidogrel being a common drug for the prevention and treatment of thrombosis (6–9). Clopidogrel is an antiplatelet drug that is converted to bioactive metabolites under the influence of cytochrome P450 isoenzymes. These metabolites irreversibly inhibit ADP binding to the platelet surface purinergic receptor P2Y_12_, thus preventing platelet activation and aggregation (6, 10, 11). The cell-based model of coagulation outlines the pivotal role played by activated platelets in global hemostatic function (12). Activated platelets act as a scaffold upon which the prothrombinase complex must form to generate thrombin. Thrombin is the predominant regulator of coagulation, further activating platelets, catalyzing the conversion of fibrinogen to fibrin, and inhibiting fibrinolysis (12). Thrombin generation (TG) assays quantify the amount of thrombin generated following *in vitro* activation, thereby providing insight into the cumulative impact of platelet and clotting factor function in an individual patient. Thrombin generation assays have been evaluated in dogs (13, 14) and are decreased following heparin and rivaroxaban administration (15–17). Rodent models demonstrate reduced TG following clopidogrel administration as well (18) but clopidogrel inconsistently attenuates TG in people (19–22). There is currently a knowledge gap regarding the impact of pharmacologic platelet inhibition on TG assays in dogs.

In most instances, clopidogrel appears to be safe and well tolerated in dogs at standard doses without routine therapeutic monitoring (6–11). Clopidogrel resistance describes a reduction in the antithrombotic effect of the drug despite appropriate dosing. Clopidogrel resistance has been described in people, but its prevalence in small animals is less well characterized (23, 24). Recently, genetic polymorphisms in cats have been identified that contribute to individual variation to clopidogrel (25). Individual variation to clopidogrel dosing was also noted in some dogs with protein losing nephropathy (26). It therefore might be prudent to undertake therapeutic monitoring in some patients, to prevent overdosing and adverse bleeding events, as well as underdosing and failure to achieve an antithrombotic effect (27). Several *ex vivo* laboratory methods can be employed to monitor the antithrombotic effects of clopidogrel (28, 29). Traditionally, flow cytometry and turbidimetric light transmittance aggregometry (LTA) have been considered the gold standard. These techniques have several limitations for veterinary practitioners, however. They are usually restricted to the research setting, since they require specialist equipment and technical expertise (e.g., preparation of small volumes of platelet rich plasma) (29, 30). Light transmittance aggregometry also fails to mimic *in vivo* physiologic conditions, since it examines platelet function in the absence of erythrocytes. Whole blood impedance aggregometry techniques offer more physiologic conditions for platelet function assessment (29, 31). This technique has been evaluated in small animals (32–35) but machines and reagents are currently unavailable to veterinary practitioners. Viscoelastic coagulation testing (e.g., thromboelastography [TEG]) has also been thoroughly investigated in small animals (36, 37). A proprietary modified TEG protocol (TEG Platelet Mapping^™^ assay [TEG-PM]) enables specific monitoring of the platelet inhibitory effects of clopidogrel (11, 38–42). Viscoelastic testing has also largely been restricted to specialty hospitals and laboratories however (43), meaning few veterinary practitioners would have access to TEG-PM for clopidogrel therapeutic monitoring.

Thrombin generation assays therefore could offer a novel alternative for the laboratory monitoring of the platelet inhibitory effects of clopidogrel in dogs. It also has potential to be more widely accessible to the general practitioner. While these assays are restricted to specialized reference laboratories, the test is performed on citrated plasma samples that can be submitted to these laboratories (44). While these tests are therefore not suitable for point-of-care monitoring, a turnaround time of a few days would be acceptable for therapeutic monitoring in dogs chronically administered clopidogrel. The main objective of this pilot study therefore was to assess TG assay in dogs before and after the administration of clopidogrel as a potential novel therapeutic monitoring option, compared to a validated clinical test, TEG-PM. We hypothesized that TG would decrease in response to clopidogrel administration, serving as a clinical correlate to platelet inhibition. A secondary aim was to assess the correlations between the TEG-PM variable MA-ADP, reflecting the ADP contribution to clot formation, and the three TG assay variables: lag time, peak, endogenous thrombin potential [ETP]. We hypothesized that changes in MA-ADP would correlate with these TG assay variables.

## Materials and methods

### Study design

A prospective experimental pilot study was designed involving healthy dogs. In each dog, a TG assay and TEG-PM were measured prior to initiating clopidogrel and again after 7 days of clopidogrel administration. The protocol was approved by the Institutional Animal Care and Use Committee at NC State University (Protocol 20-432).

### Animals

Blood samples were obtained from eight healthy, desexed purpose-bred mix-breed dogs maintained by the Laboratory Animal Resources service at NC State University. Dogs were eligible for inclusion if they were deemed healthy based on physical examination performed by a veterinarian and not receiving long term medications other than routine preventatives. Each dog then had a complete blood count, serum biochemistry profile, coagulation panel (prothrombin time, activated partial thromboplastin time, D-dimers, fibrinogen concentration, and platelet count), and a voided urinalysis collected. Each dog was maintained in their usual housing and fed commercial dog food throughout the study. Individual dogs were temporarily moved to an exam room adjacent to their housing to obtain blood samples for the study as described below.

### Blood sampling

Baseline blood samples were collected from each dog prior to the administration of clopidogrel (Day 1). Blood samples were collected via direct atraumatic venipuncture of the jugular, cephalic, or saphenous veins using a 21-gauge needle and evacuated plastic tubes. Blood was then transferred into plastic tubes containing potassium EDTA, lithium heparin, or 3.2% sodium citrate (giving a ratio of 1:9 citrate:whole blood). These samples were used for platelet count, TG, and TEG-PM as described below.

Following blood collection, each dog was then administered 20 mg clopidogrel PO once daily for a total of 7 consecutive days. On Day 7, blood samples were collected 2-h after clopidogrel administration utilizing the same venipuncture technique described above. These blood samples were used for determination of post-treatment platelet count, TG, and TEG-PM. Following venipuncture, dogs were monitored for the next hour in case of bleeding, and then returned to their normal housing without further involvement in the study.

### Laboratory evaluation

Complete blood counts prior to study enrolment and platelet counts on days 1 and 7 were performed on EDTA anticoagulated blood sample using an automated hematology analyzer in the clinical pathology laboratory of NC State University.^1^ Presence of platelet clumps was confirmed by blood smear evaluation performed by a laboratory technician. Serum biochemistry,^2^ coagulation panel,^3^ and voided urinalyses^4^ were also performed at the clinical pathology laboratory prior to study commencement.

During the study, an aliquot of the citrated whole blood samples was centrifuged within 20 min of collection at 4000 × *g* for 15 min to yield platelet poor plasma (PPP) at room temperature, which was then stored at −80°C for later batch measurement of TG. These samples were shipped overnight on dry ice and measured within 3 months of collection at Cornell University. After thawing the frozen citrated PPP, a TG assay was performed by the calibrated automated thrombogram method using a dedicated spectrofluorimeter.^5^ Samples were activated using the manufacturer’s thromboplastin reagent containing 1 pM tissue factor. Replicate assays were performed according to the manufacturer’s instructions in microtiter plates in reaction mixtures containing 80 μL test plasma (diluted 1:2 in imidazole buffered saline) combined with 20 μL of the activating reagent and 20 μL of a fluorogenic substrate/calcium trigger reagent. The TG assay measurements for each sample were calibrated against reactions run in parallel that contained the test plasma and a thrombin α2 macroglobulin complex reagent. Three TG assay parameters were calculated by the thrombinoscope software^6^: lag time (min), peak thrombin (nM), and overall endogenous thrombin potential (ETP) (nM*min) representing the area under the curve.

The remaining citrated whole blood and heparinized whole blood samples were used for TEG-PM. Analyses were performed 30 min after sample collection, with blood being left non-agitated at room temperature (approximately 23°C). The TEG-PM was performed using a proprietary testing kit in accordance with manufacturer’s recommendations using two computerized TEG analyzers.^7^ All reagents were reconstituted just prior to use. After the 30-min rest period, 1 mL of citrated whole blood was gently mixed with kaolin. Then, 340 μL of this mixed sample was added to a standard TEG pin and cup containing 30 μL of calcium chloride that was pre-warmed to 37°C in the first TEG channel. This standard kaolin activated TEG determines clot strength, as assessed by maximum amplitude of the viscoelastic tracing, due to the action of thrombin (TEG MA Thrombin). The contribution of cross-linked fibrin to clot formation (TEG MA fibrin) was assessed in the second TEG channel by adding 10 μL of the proprietary activator (Activator F), consisting of a reptilase containing reagent (that induces cleavage of fibrinogen to fibrin), and Factor XIII to 360 μL of heparinized whole blood. Finally, 10 μL of Activator F and 360 μL of heparinized blood were added to the third TEG channel along with 10 μL of ADP to yield a final ADP concentration of 2 μmol/L. This enabled determination of the contribution of ADP to clot formation (TEG MA ADP). The extent of platelet inhibition, measured as a percentage, in response to clopidogrel administration was then calculated using the equation in the TEG software:

$$100-\left[\begin{array}{l} \left(TEG\;MA\;ADP-TEG\;MA\;fibrin\right)/\\ \left(TEG\;MA\;thrombin-TEG\;MA\;fibrin\right)\;x\;100 \end{array}\right]$$

### Statistical analysis

A sample size calculation was not performed in this investigation, since this project represented a pilot study investigating a novel use of established therapeutic monitoring techniques. Descriptive data is reported as mean +/− standard deviation (SD). Parametric analysis was used to analyze the data with normal parametric distribution being assumed. Comparison of TEG-PM AND TG variables measured on Day 1 and Day 7 were made with a paired *t*-test. In addition, correlations TEG-PM MA-ADP and TG variables (Lag time, Peak, ETP) results were made using the Pearson’s correlation coefficient. Significance threshold was set at a *p*-value <0.05. Statistical analyses were performed using commercial software.^8^

## Results

Two of the eight dogs that were screened for inclusion in the study were excluded after identifying moderate thrombocytopenia. The six remaining dogs were then included in the pilot study. The mean +/− SD bodyweight of the dogs was 22.3 +/− 2.5 kg, with 4 castrated male and 2 spayed female dogs. Their mean +/− SD age was 25 +/− 1 months. Pre-study laboratory evaluation identified mild thrombocytopenia in 1 of the 6 dogs (platelet count: 189 × 0^3^/μL with platelet clumps on blood smear; reference interval [RI]: 190–468 × 10^3^/μL) and mild prolongation in aPTT in another (15.8 s, RI: 10.5–15.1 s). Neither abnormality was considered clinically relevant and did not preclude study enrolment.

The mean +/− SD dose of clopidogrel administered was 2.3 +/− 0.3 mg/kg PO q24 hours. A summary of laboratory variables (platelet count, TG assay variables, and TEG-PM parameters) is summarized in Table 1. There was no significant difference in platelet count between Day 1 (228 +/− 47 × 10^3^/μL) and Day 7 (237 +/− 40, *p* = 0.25). On day 1, mean +/− SD TG assay variables were: lag time (1.8 +/− 0.2 min), peak (76 +/− 7 nM), and ETP (399 +/− 27 nM*min). On day 7, lag time (1.8 +/− 0.2 min, *p* = 0.42), peak (72 +/− 10 nM, *p* = 0.49), and ETP (392 +/− 32 nM*min, *p* = 0.49), indicating no significant change in any TG variables after initiating clopidogrel. At baseline, the mean +/− SD TEG MA-ADP was 19 +/− 8 mm, and platelet inhibition (% inhibition) on TEG-PM was 58 +/− 27%. On day 7, there was a significant decrease in MA-ADP (9 +/− 6 mm, *p* = 0.04) and significant increase in TEG-PM % inhibition (99 +/− 3%, *p* = 0.02) corresponding with a positive antiplatelet response to clopidogrel.

**Table 1:** Mean +/− SD laboratory parameters (platelet count, TG assay variables, and TEG-PM variables), on Day 1 and Day 7 of the pilot study.

Laboratory parameter	Day 1	Day 7	*P-*value
Platelet count (x10^3^/μL)	228 +/− 47	237 +/− 40	0.25
TG assay Lag time (min)	1.8 +/− 0.2	1.8 +/− 0.2	0.42
TG assay peak (nM)	76 +/− 7	72 +/− 10	0.49
TG assay ETP (nM*min)	399 +/− 27	392 +/− 32	0.49
TEG-PM MA-Thrombin (mm)	58 +/− 4	58 +/− 3	0.99
TEG-PM MA-Fibrin (mm)	8 +/− 6	4 +/− 2	0.3
TEG-PM MA-ADP (mm)	19 +/− 8	9 +/− 6	0.04*
TEG-PM inhibition (%)	58 +/− 27	99 +/− 3	0.02*

Significance was set at *P*-value <0.05, with an * indicating statistical significance was met. ETP, endogenous thrombin potential; MA, maximum amplitude; TEG-PM, TEG Platelet Mapping^®^; TG, thrombin generation.

**Figure 1:**
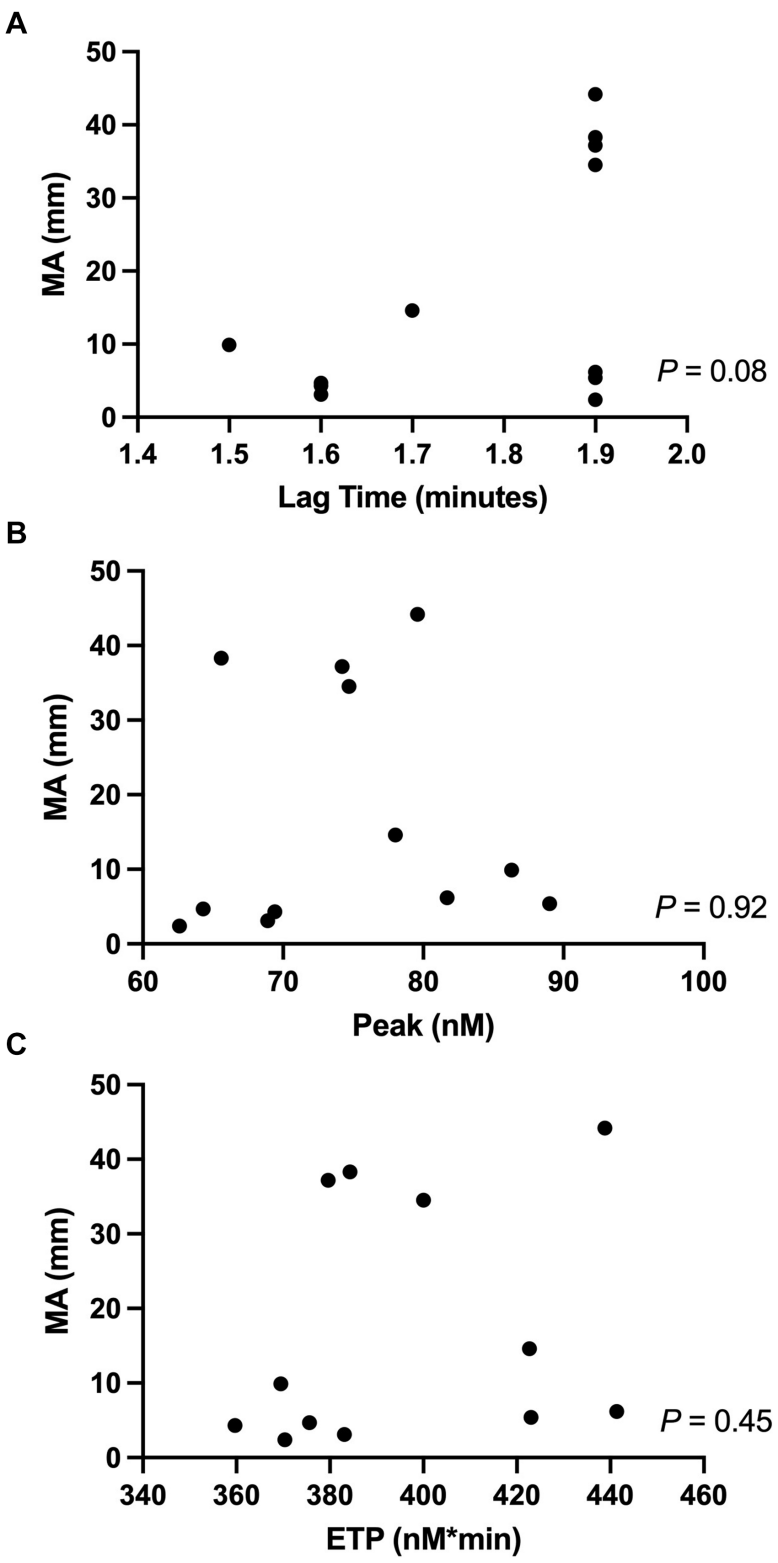
Correlations between the TEG-PM variable (MA-ADP) and TG-variables, specifically **(A)** MA-ADP (mm) vs. Lag time (min); **(B)** MA-ADP vs. Peak (nM); **(C)** MA-ADP vs. ETP (nM*min). Significance was set at a *P*-value <0.05. ETP, endogenous thrombin potential; MA, maximum amplitude; TEG-PM, TEG Platelet Mapping^®^; TG, thrombin generation.

Correlations between the TEG MA-ADP and the TG assay variables (lag time, peak, and ETP) are shown in Figure 1. No significant correlations were identified between any of the variables.

## Discussion

This pilot study set out to evaluate the potential diagnostic utility of TG assays for monitoring the antiplatelet effects of clopidogrel in dogs. Veterinary practitioners lack a readily available, convenient laboratory based therapeutic monitoring option in dogs for this purpose. Thrombin generation assays could represent a novel means of assessing the antithrombotic effect of clopidogrel in individual dogs with advantages over traditional laboratory indicators of platelet function. Despite evidence of platelet inhibition on TEG-PM, there was no significant decrease in TG in healthy dogs administered clopidogrel in this pilot study. This suggests that TG assays performed on PPP might not be an appropriate means of assessing the platelet inhibitory effect of clopidogrel in dogs. Further studies are required to understand the lack of change in TG.

Most dogs appear to tolerate clopidogrel administered at standard doses without the requirement for routine therapeutic monitoring (8, 11, 27). There is growing evidence to suggest that not all individuals respond to clopidogrel consistently, however. This is best characterized in people (23, 24), although recent work has highlighted the genetic basis of clopidogrel nonresponse in cats with hypertrophic cardiomyopathy (25). The prevalence of clopidogrel resistance in dogs is not established. In one study, most dogs with protein losing nephropathy had laboratory evidence of platelet inhibition in the face of clopidogrel administration, but one dog failed to respond in the expected way (26). Veterinary practitioners might encounter situations when monitoring the platelet inhibitory of clopidogrel in an individual dog would be desirable. This includes dogs considered at high risk for thrombotic complications (45); in dogs with persistent thrombosis despite clopidogrel administration; and to gauge bleeding risk in dogs receiving clopidogrel requiring urgent or emergent invasive procedures.

Several laboratory tests can be used to gauge the platelet inhibitory effects of clopidogrel (28–30). Flow cytometry and LTA are considered the gold standard yet are best suited for the research setting rather than clinical practice given their limited availability and technical complexity. The proprietary modified TEG assay, TEG-PM, is better suited for assessment of platelet function in clinical cases due to its standardized methodology and direct clinical application. The TEG-PM assay is based on the principle of assessing platelet function and quantifying the degree of platelet inhibition compared with maximal uninhibited platelet function. Three sets of tracings are compared to provide this information. The first tracing provides MA-Thrombin, which represents resultant clot strength under the influence of thrombin produced from a standard kaolin-activated TEG. The next tracing provides MA-Fibrin, representing the contribution of cross-linked fibrin to clot strength by the addition of reptilase and Factor XIIIa to the blood sample. Finally, MA-ADP is obtained from the third tracing, indicating the contribution of ADP to clot formation. Software within the TEG analyzer then calculates percentage inhibition as described earlier. This assay has previously been shown to correlate well with LTA in healthy dogs (11). Consequently, for the purposes of this pilot study we utilized TEG-PM as our clinical standard against which TG variables could be compared.

Major limitations to viscoelastic testing are access to equipment and the complexity of testing (43). These devices have traditionally been restricted to larger veterinary hospitals only, meaning few practitioners have easy access to TEG-PM as a method of monitoring antiplatelet drugs, especially given the short time window after blood collection where assays can be performed. Recently, a point-of-care device has been introduced to the veterinary market (46, 47). This device is reagent free, making it user friendly, but also means investigating the different contributions to clot formation is not possible. Practitioners therefore still lack a readily available laboratory test for assessing platelet function in individual dogs, hence the main objective of this pilot study being to examine TG assays as novel alternatives for therapeutic monitoring.

Endogenous hemostatic function depends on the combined and integrated function of cells and plasmatic components of the blood (12). Activated clotting factors V and X, the constituents of the prothrombinase complex, must assemble on the surface of activated platelets to generate thrombin. Thrombin in turn drives hemostatic function, by being a potent activator of platelets; catalyzing the conversion of fibrinogen to fibrin; and downregulating fibrinolysis through activation of thrombin activatable fibrinolysis inhibitor (48). Thrombin generation assays therefore enable *in vitro* estimation of the overall hemostatic potential in an individual patient (49). Applications for TG assays in people include investigation of hemorrhagic coagulopathies, monitoring replacement therapy in hemophilia, prediction of venous thromboembolism, and monitoring antithrombotic drugs (50). Thrombin generation assays have also been validated in dogs (14), with descriptions of its use in recognizing hypercoagulability in dogs with hyperadrenocorticism (13) and as a therapeutic monitoring option for anticoagulants (15–17).

The utility of TG assays for monitoring the platelet inhibitory effect of clopidogrel in dogs has not been previously investigated however. Because clopidogrel-mediated inhibition of platelets may limit the number of available phospholipid layers for clotting factors to assemble upon, the prothrombinase complex may not form and therefore limit thrombin generation. Thrombin generation assays therefore could provide indirect assessment of platelet inhibition through quantification of a vital end-product of intrinsic hemostasis. The major advantage of TG assays for practitioners would be the ability to submit blood samples to central reference laboratories without the need to maintain specialized coagulation equipment in their own practices. A turnaround time of 1–2 days would preclude its use for urgent decision making (e.g., dogs receiving clopidogrel that require unexpected emergency surgery) but would be acceptable for dogs receiving clopidogrel on a long-term basis. Thrombin generation has been shown to decrease in rats administered clopidogrel (18), but inconsistent results have been demonstrated in people receiving clopidogrel (19–21).

The results of our pilot study did not identify significant decreases in thrombin generation in this cohort of dogs administered clopidogrel despite changes in TEG-PM variables suggesting an antiplatelet effect had been achieved. This suggests TG assays performed on PPP samples are insensitive for assessing the connection between ADP-mediated platelet activation and thrombin generation. Hence, TG assays utilizing PPP might not be a laboratory therapeutic monitoring option for dogs receiving clopidogrel. The reason for TG assay poor performance might reflect an inherent lack of assay specificity for platelet function since TG is also determined by clotting factor activity. Pre-analytical factors and analytical factors might also impact the results observed leading to amplified thrombin generation *in vitro* (50). For example, fragmented platelets could have arisen from freeze–thaw cycling of samples, liberating additional phospholipid layers upon which clotting factors could assemble, hence enhancing thrombin generation. In addition, tissue factor is used as a procoagulant in the TG assay, which could have activated coagulation so strongly that any impact of platelet inhibition on thrombin generation was overwhelmed. Likewise, the activating agents used in the assay are micelles consisting of phospholipids, which could have negated the antiplatelet effect of clopidogrel in the samples. Alternatively, individual variation in the ability to generate thrombin has been reported in people, which has been attributed to difference in platelet binding proteins (51).

Limitations of this investigation include its small sample size and lack of control group consistent with the exploratory nature of a pilot study. Despite this, we believe the data demonstrating no significant changes occurring in TG assay variables, despite significant changes in TEG-PM variables, is interesting. The small sample size could mean the lack of difference in TG results was due to type II error, instead of an insensitivity of the TG assay as previously suggested. Evaluation of a larger group of dogs could be considered to verify if TG variables performed on platelet poor plasma fail to change in response to clopidogrel, especially if comparisons were made to alternative techniques (e.g., use of platelet rich plasma). A control group could be considered to further understand the results of this pilot study, for example comparison of TEG-PM and TG in healthy dogs administered placebo for 7 days. The authors however belief that the lack of a control group is not critical for interpretation of the results of this study, since predictable changes in TEG-PM occur in dogs administered clopidogrel (11). Another limitation is the choice to compare TG assays to the clinical standard of TEG-PM, rather than flow cytometry or LTA. The authors purposefully chose a comparative laboratory testing option that could plausibly be used by a clinician in practice, rather than relying on the gold standard techniques more commonly used in the research setting. Previous work also identified TEG-PM results correlate with LTA (11). Consequently, the authors feel comfortable that changes in TEG-PM variables noted here reflect platelet inhibition due to clopidogrel administration. Finally, some dogs in this study demonstrated some evidence of mild platelet inhibition on TEG-PM at baseline, prior to the administration of clopidogrel. The reason for this is unclear but the potential for genetic differences in platelet receptor function could be implicated.

## Conclusion

In summary, the present pilot study investigated a small group of dogs that had TEG-PM and TG assays performed prior to and after 7 days of clopidogrel administration. After 7 days of clopidogrel administration, TEG-PM assays showed changes consistent with a platelet inhibitory effect. In contrast, TG assays in platelet poor plasma did not differ from pre-treatment values. Thrombin generation assays performed on PPP do not appear to be an appropriate means of monitoring the antiplatelet effect of clopidogrel in dogs based on this work. Further studies evaluating the reason or lack of change in TG assays, as well as the prevalence of clopidogrel resistance in dogs would be beneficial. This could help enlighten veterinarians about the relative risk of encountering dogs that fail to respond to clopidogrel appropriately, as well as the potential to recognize if certain groups of dogs are at a greater risk. This would help guide whether post-treatment antiplatelet monitoring would be recommended. It is also prudent to continue to search for accurate, clinically user-friendly laboratory tests and define recommended therapeutic monitoring strategies for dogs receiving antithrombotic drugs.

## Data availability statement

The original contributions presented in the study are included in the article/supplementary material, further inquiries can be directed to the corresponding author.

## Ethics statement

The animal study was approved by North Carolina State University. The study was conducted in accordance with the local legislation and institutional requirements.

## Author contributions

KR: data acquisition, data analysis, and manuscript preparation. AL: study idea, study design, data acquisition, data analysis, and critical revision. LR: study design, data acquisition, analysis, and critical revision. RL: data analysis and critical revision. YU: study design, data acquisition, data analysis, statistical analysis, and critical revision. All authors contributed to the article and approved the submitted version.

## Funding

This study was funded by a Seed Incentive Fund at NC State University, College of Veterinary Medicine.

## Conflict of interest

The authors declare that the research was conducted in the absence of any commercial or financial relationships that could be construed as a potential conflict of interest.

The reviewer CB declared a past co-authorship with the author AL to the handling editor.

## Publisher’s note

All claims expressed in this article are solely those of the authors and do not necessarily represent those of their affiliated organizations, or those of the publisher, the editors and the reviewers. Any product that may be evaluated in this article, or claim that may be made by its manufacturer, is not guaranteed or endorsed by the publisher.

## Footnotes

^1^Bayer ADVIA 120, Siemens Medical Solutions USA, Inc., Malvern, PA.

^2^Stago Compact, Diagnostica Stago, Parsippany, NJ.

^3^STA Compact, Diagnostica Stago, Parsippany, NJ.

^4^Roche U411 Analyzer, Roche Diagnostics, Indianapolis, IN.

^5^Thrombin Calibrator, Diagnostica Stago, Parsippany, NJ.

^6^Thrombinoscope Software, Diagnostica Stago, Assieres, France.

^7^RapidTEG, Haemonetics Corp, Braintree, MA.

^8^GraphPad Prism Version 9.3.0, San Diego, CA.

## Abbreviations

ETP: endogenous thrombin potential
LTA: light transmittance aggregometry
MA: maximum amplitude
PPP: platelet poor plasma
TEG: thromboelasotography
TEG-PM: thromboelastography Platelet Mapping^®^
TG: thrombin generation
